# Temporal Trends in the Incidence and Mortality of Major Reproductive-Related Cancers in Women in Guangzhou From 2010 to 2020: A Joinpoint and Age-Period-Cohort Study

**DOI:** 10.3389/ijph.2023.1605300

**Published:** 2023-03-23

**Authors:** Suixiang Wang, Shan Liu, Zhiqiang Nie, Yexin Li, Ke Li, Huanzhu Liang, Qian Chen, Lin Wen, Huan Xu, Boheng Liang, Pengzhe Qin, Chunxia Jing

**Affiliations:** ^1^ Guangzhou Center for Disease Control and Prevention, Guangzhou, China; ^2^ Department of Public Health and Preventive Medicine, School of Medicine, Jinan University, Guangzhou, China; ^3^ Department of Cardiology, Hypertension Research Laboratory, Guangdong Cardiovascular Institute, Guangdong Provincial People’s Hospital, Guangdong Academy of Medical Sciences, Guangzhou, China; ^4^ Guangdong Key Laboratory of Environmental Pollution and Health, School of Environment, Jinan University, Guangzhou, China

**Keywords:** age-period-cohort analysis, women, joinpoint regression, reproductive-related cancers, Guangzhou

## Abstract

**Objective:** To understand the temporal trends of cancer incidence and mortality in women in Guangzhou during the past 11 years and provide clues for future research.

**Methods:** Data were obtained from the Guangzhou Cancer and Death Registry. Average annual percentage changes (AAPCs) in age-standardized incidence rates (ASIRs) and age-standardized mortality rates (ASMRs) were obtained by joinpoint regression. The age-period-cohort (APC) model was generated to quantify the effects of age, period, and cohort.

**Results:** The ASIRs for cervical (AAPC = −4.3%) and ovarian (AAPC = −3.2%) cancers showed a downward trend during 2010–2020, and that for uterine cancer showed an upward trend. The ASMRs of breast (APC = 5.0%) and cervical (APC = 8.8%) cancers increased. The APC model highlights different age, period, and birth cohort effects depending on the cancer site.

**Conclusion:** The ASIRs for cervical and ovarian cancers among women in Guangzhou showed a decreasing trend during the period. The APC model showed mortality for 4 cancers increased with age. Incidence and mortality decreased with increasing birth cohort. Annual reproductive cancer screening is recommended for women of appropriate age to reduce the disease burden.

## Introduction

With economic development, aging societies, and rising living standards, cancer is a major public health problem worldwide (1, 2). In 2012, malignant neoplasms were the second leading cause of death in developing countries and the first leading cause of death in developed countries (3). In China, cancer has been a significant public health problem and the leading cause of death since 2010 (4). Among the various cancers that are highly prevalent in women, breast cancer has now surpassed lung cancer as the leading cause of cancer incidence and deaths in women in most developing countries (5). Cervical cancer is unevenly distributed geographically, with a higher incidence in developing countries than developed countries (6, 7). Uterine cancer is the 6th most common neoplasm in women worldwide and the 14th leading cause of cancer-related death (8).

In southern China, Guangzhou has become one of the world’s most prosperous metropolises. An increasing number of people are moving to Guangzhou for better living conditions, and the population of Guangzhou is growing year by year, including a mechanical growth of 226,100 people in 2020 (9). The women’s household population also increased from 3,971,485 in 2010 to 4,892,561 in 2020 (10). As the second leading cause of death in Guangzhou, cancer is a major public health challenge in the city (11). During 2014–2015, the incidence of malignant tumors in Guangzhou reached 299.10 per 100,000, and the mortality rate was 158.28 per 100,000 (12). The 2015–2016 annual report of the Guangzhou Oncology Registry showed that the incidence of cancer in women rose to 238.82 per 100,000 in 2013. The mortality rate of malignant neoplasms in women reached 111.36 per 100,000.

In 2012, breast cancer became the leading cancer in women, while cervical, uterine, and ovarian cancers were included in the top 10 cancers in women. These 4 cancers have also been included in the top 10 causes of cancer death in women (13). Women are playing an increasingly important role in society. On the one hand, an increasing number of women are changing their traditional attitudes, participating in social work, and taking on essential responsibilities to promote social development. On the other hand, women also have the difficult task of bearing children. Therefore, reducing the burden of cancer on women and understanding the situation and trends in cancer among women are important not only for health but also for addressing sex disparities and recognizing women’s participation in socioeconomic development and as caregivers in the family (14).

Joinpoint regression analysis is widely used as a valuable tool to investigate trends in incidence and mortality over time (15, 16). The age-period-cohort (APC) model is a valuable tool that has the advantage of dividing temporal variation into three dimensions to assess the effect of these three factors on disease incidence or mortality (17–19).

In this study, we analyzed the trends in the incidence and mortality rates of 4 common reproduction-related cancers (breast, cervical, uterine, and ovarian) in the women’s household population of Guangzhou over time from 2010 to 2020 using joinpoint regression models. An age-period-cohort model was used to estimate the trend effects of age, period, and cohort on the incidence and mortality of these 4 cancers. We aimed to provide a database for policymakers to specify future targeting of these 4 reproduction-related cancers in women.

## Methods

### Data Source

In this study, we collected data on incident cases and deaths due to cancer in Guangzhou from 2010 to 2020, with the breasts, cervix, uterus, and ovaries as the primary cancer sites. Eleven administrative districts in Guangzhou were covered, including Zengcheng, Yuexiu, Tianhe, Nansha, Liwan, Huangpu, Huadu, Haizhu, Panyu, Conghua, and Baiyun Districts. Patients with non-Guangzhou household registration and without complete information on sex, age at diagnosis, race, and marital status were removed. The incidence rate formula was as follows: (The number of new cases of a disease in a population during a certain period/the number of people exposed during the same period) *K. The annual Guangzhou women’s household population data in the same period of analysis were obtained from the Guangzhou Bureau of Statistics. Our study complied with the Guidelines for Accurate and Transparent Health Estimates Reporting (GATHER) statement (Annex 1). Due to the retrospective nature of this study and the anonymity of personal information, the requirement for ethics committee approval was waived.

### Inclusion Criteria

The number of new cases or deaths from cancer among women aged 20 years or older with household registration in Guangzhou from 2010 to 2020 was determined. All patients with primary cancer or carcinoma *in situ* were judged by the following ICD-10 codes: Breast cancer: C50, C50.0, C50.1, C50.2, C50.3, C50.4, C50.5, C50.7 and C50.8; *In situ* breast cancer: D50, D50.7, and D50.9; Cervical cancer: C53.0, C53.1, C53.8 and C53.9; cervical carcinoma *in situ*: D06.1, D06.7 and D06.9; and uterine cancer: C54.0, C54.1, C54.2, C54.3, C54.8, C54.9, C55.9. Ovarian cancer codes (C56.0 and C56.9) were included in the database (20).

### Exclusion Criteria

Patients who were not residents of Guangzhou, patients aged less than 20 years, patients with incomplete information on sex, age at diagnosis, race, and marital status, and patients with duplicate information on incidence or mortality were excluded.

### Statistical Analysis

EXCEL and Stata 14.0 statistical software were used to organize and analyze the data of each group. Cancer incidence and mortality rates were calculated for cancer site and age group (5-year age groups) stratification for 2010 to 2020. Age-standardized incidence rates (ASIRs) and age-standardized mortality rates (ASMRs) were calculated based on 2010 census data in China.

Joinpoint regression was used to analyze the incidence and mortality trends of 4 cancers among women in Guangzhou from 2010 to 2020 using joinpoint regression program software version 4.9.0.0 (National Cancer Institute) (21). The basic principle of joinpoint regression is to identify the inflection points of the model, that is, the position and number of joinpoint points, and divide the long-term trend of incidence or mortality of the time series into several segments between the inflection points. The method generates the annual percent change (APC) and the estimated average annual percent change (AAPC) (22). By analyzing whether the values of APC and AAPC are statistically significant, the change trend of related morbidity or mortality can be obtained. An age-period-cohort study was used to analyze the association between ages, periods and cohorts and cancers in women. The APC model pooled data for 14 5-year age groups (from 20–24 years to 85+ years) and 11 one-year periods (consecutive years from 2010 to 2020) for cases and populations. The Poisson log-linear model modeled the incidence and mortality rates for the 4 female reproduction-related cancers. The Poisson log-linear model is commonly used in epidemiology, assuming that the counts of incidence and mortality events for the 4 cancers follow a Poisson distribution and that the log-linear model estimates the incidence and mortality rates. The model can be expressed as:

$$\log r_{ij}=\mu +\alpha_{i}+\beta_{j}+\gamma_{k}$$

where r_ij_ denotes incidence and mortality at age group $i\;\left(i=1\ldots ,a\right)$ and period year $j\;\left(j=1,\ldots .,p\right)$ ; μ denotes the overall global mean; α_i_ denotes the effect of age group *i* (a = 14); β_j_ denotes the effect of exam year *j* (*p* = 11); and γ_k_ denotes the effect of cohort *k* (23).

Since the incidence rate of the 4 cancers in women under 20 years of age was too small to be included in the analysis, our incidence rate includes women over 20 years of age. For mortality, we removed data with 4 or more years of missing mortality data in the 2010–2020 age group for model fitting.

To interpret our results visually, we calculated the rate ratio (RR) (24, 25) as the exponential value of the regression coefficient. It refers to the ratio of women’s cancer risk to the overall mean rate for a given age group, period, or cohort. The analysis was implemented by the APCG1 package (26) in R.

## Results

A total of 44,044 cancer cases and 10,373 cancer deaths between 2010 and 2020 were included in the study, including 26,460 cases of breast cancer and 5,371 deaths, 7,025 cases of cervical cancer and 1,863 deaths, 6,739 cases of uterine cancer and 1,362 deaths, and 3,820 cases of ovarian cancer and 1,777 deaths.

Between 2010 and 2020, the majority of cancer cases and deaths among women were among married women, with Yuexiu District having the highest incidence (Supplementary Table S1). Breast and cervical cancers in the 40–64-year age group and ovarian and uterine cancers in the 45–64-year age group accounted for more than 10% of the total number of cancer cases. The percentage of breast and uterine cancers in the 50–69-year age group, cervical cancer in the 45–64-year age group and ovarian cancer in the 50–74-year age group also exceeded 10% of the total number of deaths. The ASIR and ASMR of women in Guangzhou from 2010 to 2020 are shown in Table 1. The incidence of breast and uterine cancers in women did not change much during 2010–2020. The incidence of cervical and ovarian cancers showed a downward trend. The ASIR of cervical cancer dropped from 18.98/100,000 in 2010 to 11.75/100,000 in 2020, and the highest ASIR was 21.31/100,000 in 2013. The ASIR of ovarian cancer dropped from 10.95/100,000 in 2010 to 6.11/100,000 in 2020, with the highest ASIR (10.95/100,000) in 2010, while the ASMRs of the 4 cancers remained stable (Table 1).

**TABLE 1:** Incidence and mortality of reproductive-related cancers in women in Guangzhou 2010–2020 (per 100,000).

Year	Breast cancer				Cervical cancer				Uterine cancer				Ovarian cancer			
	Incidence		Mortality		Incidence		Mortality		Incidence		Mortality		Incidence		Mortality	
	Crude rate	ASIR^a^	Crude rate	ASMR^a^	Crude rate	ASIR	Crude rate	ASMR	Crude rate	ASIR	Crude rate	ASMR	Crude rate	ASIR	Crude rate	ASMR
2010	62.39	62.53	10.13	9.76	18.58	18.98	3.43	3.47	14.84	14.78	2.64	2.55	10.97	10.95	3.36	3.16
2011	64.81	64.57	12.60	12.11	18.60	18.89	3.24	3.20	16.37	16.23	3.65	3.40	10.69	10.57	3.83	3.61
2012	63.96	63.23	11.90	11.23	18.24	18.31	3.44	3.33	16.35	16.05	3.99	3.74	10.38	10.14	4.51	4.18
2013	74.60	72.23	13.13	12.12	21.45	21.31	4.19	3.97	17.41	16.82	3.65	3.35	10.35	9.97	4.43	4.03
2014	73.91	71.11	12.88	11.62	20.34	19.85	5.14	4.73	19.49	18.58	2.79	2.53	9.85	9.32	4.06	3.64
2015	68.80	65.75	14.75	13.14	17.67	17.13	4.83	4.32	18.65	17.54	3.30	2.82	10.64	10.06	4.71	4.16
2016	66.70	62.99	15.63	14.19	18.55	17.57	6.07	5.52	18.12	16.92	3.40	2.99	11.20	10.65	5.56	4.74
2017	73.38	68.47	15.67	13.70	21.99	21.24	6.43	5.89	17.97	16.73	3.41	2.95	9.98	9.37	4.88	4.24
2018	74.00	69.11	16.11	13.62	17.22	16.17	5.26	4.64	17.79	16.51	3.83	3.24	8.74	8.13	5.02	4.31
2019	73.06	67.13	14.86	12.67	16.49	15.28	5.45	4.65	18.76	17.14	4.22	3.56	10.33	9.42	5.56	4.69
2020	60.95	55.91	15.03	12.42	12.87	11.75	5.28	4.38	16.72	15.11	3.92	3.29	6.77	6.11	4.62	3.74

^a^ ASIR, age-standardized incidence rate of the Chinese population; ASMR, age-standardized mortality rate of the Chinese population.

The joinpoint regression results for the ASRs in the incidence and mortality of the 4 cancers in Guangzhou from 2010 to 2020 are shown in Table 2. The average annual change percentage (AAPC) of the ASIR for breast cancer in Guangzhou was −0.36%, but no significant difference was observed between 2010 and 2020. The ASIR trend of cervical cancer decreased significantly from 2017, with an APC = −14.25% (−25.9%-0.8%). During 2010–2014, the ASIR of uterine cancer increased, with an APC = 4.9% (0.4%–9.5%), but during 2014–2020, it decreased, with an APC = -2.2% (−4.3%∼-0.2%). The ASIR of ovarian cancer decreased from 2010 to 2020, with an APC = −3.2% (−5.6%∼−0.8%). The ASMR of breast cancer increased from 2010 to 2016, with an APC = 5.0% (1.5%–8.7%). The ASMR of cervical cancer showed a statistically significant upward trend before 2017, with an APC = 8.8 (4.6–13.3). There was no significant difference between the ASMR APC and AAPC in women with uterine and ovarian cancers in Guangzhou.

**TABLE 2:** Joinpoint regression analysis of reproductive-related cancer incidence and mortality trends in women in Guangzhou 2010–2020.

Cancers	Rate	AAPC (%,95%CI)	Trend 1		Trend 2	
			Period	APC (%,95%CI)	Period	APC (%,95%CI)
Breast cancer	ASIR	−0.4 (−2.0∼1.3)	2010–2020	−0.4 (−2.0∼1.3)		
	ASMR	1.9 (−0.6∼4.4)	2010–2016	5.0*(1.5∼8.7)	2016–2020	−2.6 (−7.9∼2.9)
Cervical cancer	ASIR	−4.3*(−8.1∼−0.3)	2010–2017	0.4 (−3.3∼4.1)	2017–2020	−14.3*(−25.9∼−0.8)
	ASMR	3.1 (−0.7–7.1)	2010–2017	8.8*(4.6–13.3)	2017–2020	−9.1 (−20.0∼3.4)
Uterine cancer	ASIR	0.6 (−1.2–2.3)	2010–2014	4.9*(0.4∼9.5)	2014–2020	−2.2*(−4.3∼−0.2)
	ASMR	0.7 (−2.1–3.4)	2010–2020	0.7 (−2.1∼3.4)		
Ovarian cancer	ASIR	−3.2*(−5.6∼−0.8)	2010–2020	−3.2*(−5.6∼−0.8)		
	ASMR	1.9 (−0.4–4.3)	2010–2020	1.9 (−0.4∼4.3)		

* means that it is statistically significant, *p* < 0.05, APC or AAPC was significantly different from 0 (two-sided *p* < 0.05).

**FIGURE 1:**
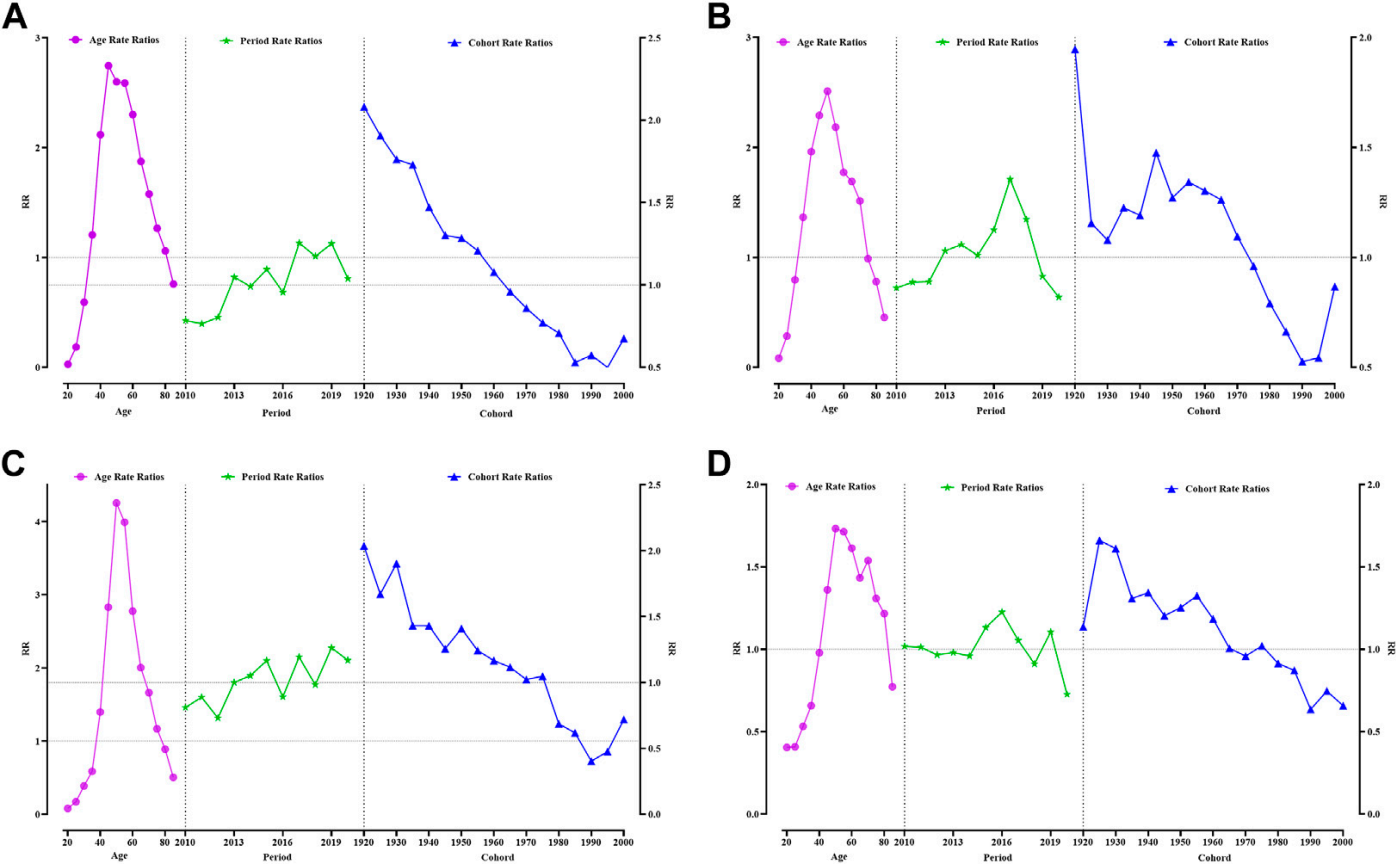
Analysis of women reproductive-related cancers incidence by APC model. **(A)** Breast cancer, **(B)** Cervical cancer, **(C)** Uterine cancer, **(D)** Ovarian cancer. Guangzhou 2010–2020.

In the age-period-cohort model, age was significantly associated with the incidence of the 4 cancers (Figure 1). An age between 35–84 years was an effect factor for breast cancer incidence. The age effect for breast cancer incidence increased sharply from 35 to 54 years of age and decreased slowly after 55 years of age. The highest effect values for breast cancer occur at ages 45–49 years, with a 2.75-fold increase in the effect of developing breast cancer. Between 2010 and 2020, breast cancer was diagnosed with an increased period effect between 2017 and 2019 compared with 2014 (RR (95% CI) > 1, *p* < 0.05). The cohort effect for breast cancer incidence declined progressively with later birth cohorts. The cohort effect for breast cancer incidence was greatest for women born in 1920–1924 (RR (95% CI) = 2.08 (1.89–2.30), *p* < 0.05) compared to those born in 1965–1969 (Figure 1A, Supplementary Table S2).

The age effect for cervical cancer incidence increased sharply from 35 to 54 years of age and decreased slowly after 55 years of age, such as that for breast cancer. The highest effect factor for the development of cervical cancer occurred in the 50–54-year age group. Women in this age group have a 2.51-fold increased risk of developing cervical cancer compared to those at approximately 33 years of age. The period effect for cervical cancer incidence was most significant in 2017, with an effect coefficient for the period of 0.35 (RR (95% CI) = 1.35 (1.26–1.46), *p* < 0.05). The incidence of cervical cancer fluctuated more among women born before the 1970s, with the two highest effect birth years being 1920–1924 (RR (95% CI) = 1.95 (1.66–2.28), *p* < 0.05) and 1955–1959 (RR (95% CI) = 1.34 (1.22–1.48), *p* < 0.05) (Figure 1B, Supplementary Table S3).

The age effect for uterine cancer was approximately 5 years later than that for breast and cervical cancers, with the effect of uterine cancer beginning in the 40–44-year age group and the peak effect occurring in the 50–54-year age group with an effect coefficient = 1.45 and RR (95% CI) = 4.25 (3.90–4.64). The period effect for uterine cancer incidence was greatest in 2019 (RR = 1.26) compared with 2014. The cohort effect coefficient for uterine cancer was greater than 0.5 for women born between 1920 and 1934, with the maximum effect year being 1920–1924 (RR (95% CI) = 2.04 (1.75–2.36), *p* < 0.05). The effect of ovarian cancer was higher for women born between 1925 and 1964 (RR > 1, *p* < 0.05) (Figure 1C, Supplementary Table S4).

The age group with the highest effect of ovarian incidence was 55–59 years, with an effect coefficient = 0.54 (RR (95% CI) = 1.71 (1.55–1.90), *p* < 0.05). The period effect for uterine cancer incidence was most remarkable in 2019 (RR = 1.26) compared with 2014 (Figure 1C, Supplementary Table S4). For ovarian cancer, the year with the highest period incidence effect factor was 2016 [RR (95% CI) = 1.23 (1.13–1.33), *p* < 0.05]. The cohort effect coefficient for ovarian cancer gradually decreased as the birth cohort moved backward from 1925 to 1929, [RR (95% CI) = 1.66 (1.45–1.91)] (Figure 1D, Supplementary Table S5).

**FIGURE 2:**
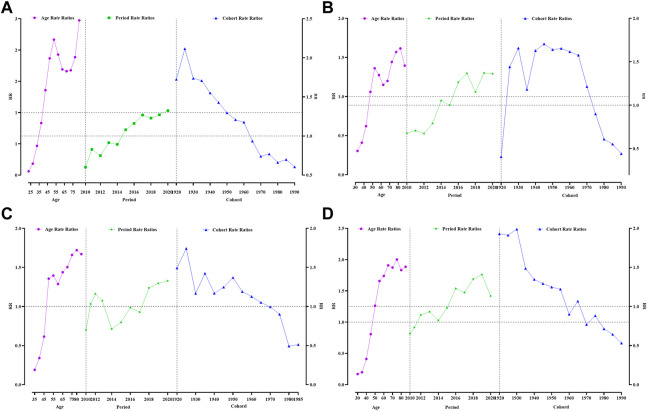
Analysis of women reproductive-related cancers mortality by APC model. **(A)** Breast cancer, **(B)** Cervical cancer, **(C)** Uterine cancer, **(D)** Ovarian cancer. Guangzhou 2010–2020.

Age, period and cohort had different effects on the 4 types of reproductive cancer deaths among women in Guangzhou. The age group effect for death from breast cancer was younger than that for the other three cancers, with an increased effect of death from breast cancer in women aged 45–49 years and above and a fluctuating increase in the age effect, with the largest age group effect occurring at 85 years and above [RR (95% CI) = 2.47 (2.29–2.68)]. The results showed that the period effect for breast cancer increased from 2015, with a gradual increase in the effect coefficient over the period, from 0.08 in 2015 to 0.28 in 2020. Women born between 1925 and 1929 had the highest effect factor of 0.75 (Figure 2A, Supplementary Table S6).

The highest age effect for death from cervical cancer was 80–84 years, with an effect coefficient = 0.48 [RR (95% CI) = 1.62 (1.48–1.76)]. The mortality cohort effect for cervical cancer increased and then decreased from 1920 onward, with the highest cohort effect being 1945–1949 [RR (95% CI) = 1.71 (1.55–1.79)] (Figure 2B, Supplementary Table S7).

The highest age effect for death from uterine cancer was 80–84 years, with an effect coefficient = 0.54 [RR (95% CI) = 1.72 (1.53–1.94)]. The period effect of death from uterine cancer appeared the latest and only started to increase in 2018. Those born between 1925 and 1929 had the highest effect factor of 0.55 (RR > 1, *p* < 0.05) (Figure 2C, Supplementary Table S8).

The highest age effect for death from ovarian cancer was younger than that for all three previous cancers, at 75–79 years [RR (95% CI) = 2.00 (1.80–2.22)]. The highest cohort effect for ovarian cancer was 1930–1934, with a cohort effect coefficient of 0.69 [RR (95% CI) = 1.99 (1.75–2.26)] (Figure 2D, Supplementary Table S9).

## Discussion

Our study showed that the AAPC in the incidence of cervical and ovarian cancers among women in Guangzhou from 2010 to 2020 showed a decreasing trend, while the AAPC in the mortality rates of the 4 cancers tended to stabilize. In the APC model, we found that incidence and mortality rates for the 4 cancers were strongly associated with age and birth cohort, with the early birth cohort having a higher risk of cancer incidence and mortality than the late birth cohort.

This is the first study to examine the effects of the APC model on 4 common reproductive cancers in women in Guangzhou, China. Our findings were not consistent with the incidence of breast cancer in Shanghai and uterine and cervical cancers in China (27, 28), which may be related to the level of local economic development and the social security system. Compared to those from the United States, our results for breast cancer and uterine cancer incidence and mortality are lower, which is probably related to the diet of the local population (29). Nevertheless, we have a higher cervical cancer incidence and mortality than the United States (30, 31), which may be related to the timing of the introduction of the HPV vaccine in the United States and the differences in socioeconomic development between the two countries.

The joinpoint regression model allowed us to better capture pieces of the changing trends. Throughout the study period, the incidence of uterine cancer reached an inflection point in 2014. The inflection point for cervical cancer incidence occurred in 2017, with incidence rates showing an upward and then a downward trend. In contrast, the incidence of ovarian cancer showed a slow decline over the study period, which may be related to free screening for two cancers (cervical and breast) being provided for women at the appropriate age in Guangzhou since 2009 (32) and the improvement of the social security system in Guangzhou (33), which has helped to ensure better access to health services for women, thereby increasing cancer screening and reducing the incidence probability. Through cancer screening and health education, the health concerns of people, especially women, have increased. The improvement of Guangzhou’s social security system is relevant.

Our study showed an increase in the age-standardized mortality rates for all 4 types of cancer in Guangzhou, a phenomenon also seen in countries such as South Africa (34). Cancer deaths are influenced by many factors, such as age, smoking status, particulate air pollution exposure, physical inactivity, city living, education level and race (35, 36). With the economic development of our country, people’s economic levels have improved, but the pollution of the environment is becoming increasingly serious. Previous studies have shown that environmental pollution is severe in Guangzhou, suggesting that it may increase the disease burden of cancer (37, 38). With the increase in sedentary activities, people are increasingly less physically active, which has a very serious impact on the survival of cancer patients. Existing studies have shown that exercise can improve the survival of cancer patients (39, 40), and the lack of necessary exercise may increase the risk of cancer death.

The APC model showed that each of these three factors had a specific association with cancer incidence and death. We could not separate these three factors in relevant studies. Incidence increases significantly with age, and the risk of all 4 cancers is high during the perimenopausal period. Perimenopause lasts for 10–15 years (41). During this time, follicle stimulating hormone (FSH) levels remain elevated, whereas anti-Mullerian hormone (AMH) and inhibin B levels, as well as estrogen levels, decrease (42, 43), and sex hormone levels are associated with breast, uterine and ovarian cancers (44). The incidence and mortality risks for all 4 cancers increased with age, which is consistent with national findings and epidemiological studies in Guangzhou and Shenzhen, China (45, 46). This may be related to population aging. In 2020, the average life expectancy of the Guangzhou population was 82.9 years (47), which is higher than the national life expectancy (77.3 years) (48), suggesting that the future cancer burden will increase as the elderly population continues to grow.

The cohort effect showed that the risk of breast cancer was most significant in the earliest birth cohort. Previous studies have shown that overweight, breastfeeding, breastfeeding duration, a reduced number of children due to the one-child policy, soy product intake, late menopause, family history of breast cancer, and passive smoking are significantly associated with the risk of breast cancer in Chinese women (49–52). The risk of breast cancer incidence and death began to decline in later birth cohorts in 1965. This part of the population is in a good social environment with better economic development, social security and medical care. Reports show that the implementation rate of the 10-year plan for the development of women and children in Guangzhou (2011–2020) is 98.26%, related to the fact that by 2021, the rate of exclusive breastfeeding of infants in Guangzhou was 65.54% (53). Adherence to breastfeeding can reduce the incidence of breast cancer (54). At the same time, Guangzhou has pioneered a three-stage screening system, which has improved the sensitivity and specificity of breast cancer screening programs, better achieving “three early prevention and control” and reducing the risk of death (55, 56).

The results of the study showed that the effects of age and cohort were most pronounced in the incidence and mortality rates of cervical cancer among women in Guangzhou between 2010 and 2020. Women’s risk of developing cervical cancer increases with age. The age group with the highest risk was 45–54-year age group, which is quite different from that in the previously reported study (57). Regarding mortality, being older than 50 years and being born before 1975 are risk factors for death from cervical cancer. Possible reasons for the differences among age groups include HPV prevalence (58) and screening availability (59) between regions. The cohort effect was concentrated on those born before the 1970s. A significant risk factor for the development of cervical cancer is HPV infection (60). In a population-based study, the number of lifetime sexual partners was found to be associated with the risk of HPV infection in a Guangzhou cohort (61). With the increasing awareness of cancer prevention among women in Guangzhou and the popularity of the HPV vaccine, HPV infection can be detected and controlled early in young women, thus reducing the incidence and mortality of cervical cancer.

In Guangzhou, the risk of uterine and ovarian cancer remains highest in people aged >40 years, while the risk of death is >50, which may be related to changes in reproductive and other lifestyle factors. With economic development and increased energy intake, the obesity rate among Chinese women has increased over the past 20 years (62), and obesity is one of the risk factors for the development of uterine cancer (63). Later, with the “Healthy China 2020” program (64), people became aware of the importance of a healthy weight, which may also be an influential factor in reducing the incidence of and mortality from uterine cancer. In 1986, family planning (65) was introduced in Guangdong Province, requiring late marriage and childbirth and emphasizing fewer and better births, and women over 45 years of age experienced this period. Earlier age of menarche, later age of menopause, and fewer pregnancies all increase the risk of ovarian cancer disease and death by increasing the total ovulatory cycle (66).

In conclusion, trends in incidence and mortality rates from 2010 to 2020 are influenced by age, cohort, and period effects, reflecting the far-reaching impact of socioeconomic development and lifestyle factors. We suggest that Guangzhou districts implement free five-cancer screening for the resident population while increasing publicity efforts to promote annual reproductive cancer screening for women of the appropriate age to reduce the burden of disease.

### Limitations and Advantages

The main limitation of this study is that the research population was limited to Guangzhou, and it is difficult to generalize the results to all parts of the country. Another limitation of this study is that the time span was limited to only 11 years. There may be crossover in the evaluation of age, period, and cohort effects, and the distinction between age and period effects may not be clear. Second, epidemiological measures of morbidity and mortality were limited, and important measures such as disability-adjusted life years were missing in both subgroups. Finally, because the system did not require that the information in the form to be 100% complete, the individual clinical information we collected needed to be more comprehensive, and some patients with unclear stages might have been the subjects of our study but were excluded. Among the tumor types, we could only determine whether they were breast cancer, cervical cancer, uterine cancer, or ovarian cancer tumors according to ICD-10 codes. Nevertheless, the cancer type and location were not available, which failed to make the classification of the cancer more detailed. Therefore, in future research, we need to include more periods and different cities to verify our results, and the data collection needs to be improved. In addition, the survival of the subjects was included in the analysis to obtain more accurate effects and a more complete presentation and provide more rigorous data support for future policy-making. The main strengths of this study are the robust methodology and statistical methods used to evaluate the data and produce the results. In addition, highlighting trends in measures over the decade is another strength of the study.
